# Abdominal Contents from Two Large Early Cretaceous Compsognathids (Dinosauria: Theropoda) Demonstrate Feeding on Confuciusornithids and Dromaeosaurids

**DOI:** 10.1371/journal.pone.0044012

**Published:** 2012-08-29

**Authors:** Lida Xing, Phil R. Bell, W. Scott Persons, Shuan Ji, Tetsuto Miyashita, Michael E. Burns, Qiang Ji, Philip J. Currie

**Affiliations:** 1 Department of Biological Sciences, University of Alberta, Edmonton, Alberta, Canada; 2 Pipestone Creek Dinosaur Initiative, Clairmont, Alberta, Canada; 3 Institute of Geology, Chinese Academy of Geological Sciences, Beijing, China; Raymond M. Alf Museum of Paleontology, United States of America

## Abstract

Two skeletons of the large compsognathid *Sinocalliopteryx gigas* include intact abdominal contents. Both specimens come from the Jianshangou Beds of the lower Yixian Formation (Neocomian), Liaoning, China. The holotype of *S. gigas* preserves a partial dromaeosaurid leg in the abdominal cavity, here attributed to *Sinornithosaurus.* A second, newly-discovered specimen preserves the remains of at least two individuals of the primitive avialan, *Confuciusornis sanctus*, in addition to acid-etched bones from a possible ornithischian. Although it cannot be stated whether such prey items were scavenged or actively hunted, the presence of two *Confuciusornis* in a grossly similar state of digestion suggests they were consumed in rapid succession. Given the lack of clear arboreal adaptations in *Sinocalliopteryx*, we suggest it may have been an adept stealth hunter.

## Introduction

Abdominal contents provide the most reliable record of diet in extinct vertebrates although preservation of such remains is rare and frequently difficult to demonstrate unequivocally. The Lower Cretaceous Yixian Formation in northeastern China preserves a remarkably diverse terrestrial fauna in fine-grained volcaniclastic-lacustrine deposits [1], [2]. Such lagerstätten preserve remarkable anatomical features, including integumentary structures, organic compounds (such as proteins responsible for pigmentation), and abdominal contents in exquisite detail [3]. To date, gut contents have been found in a wide range of Jehol taxa (Table 1), demonstrating clear trophic relationships within the Jehol ecosystem.

**Table 1:** Reported abdominal contents of Jehol taxa.

Taxon		Abdominal contents	Reference
Mammalia	*Repenomamus*	*Psittacosaurus*	Hu et al. [67]
Dinosauria	*Sinosauropteryx*	unidentified mammal	Chen et al. [15]; Currie and Chen [10]
	*Sinocalliopteryx*	*Sinornithosaurus*, *Confuciusornis*, unidentified ornithiscian	Ji et al. [4]; this study
	*Microraptor*	enantiornithine bird	O’Connor et al. [54]
Aves	*Confuciusornis*	cf. *Jinanichthys*	Dalsätt et al. [68]
	*Hongshanornis*	Plant seeds	Zheng et al. [69]
	*Jeholornis*	Plant seeds	Zhou and Zhang [70]
	*Jianchangornis*	Fish remains	Zhou et al. [71]
	*Sapeornis*	Plant seeds	Zheng et al. [69]
	*Yanornis*	Fish remains	Yuan [72]; Zhou et al. [73]
Choristodera	*Hyphalosaurus*	Fish remains	UALVP 54043, Unpublished
Squamata	*Yabeinosaurus*	Fish remains	Zhou and Wang [74]

Compsognathidae, typified by the type species *Compsognathus longipes,* includes a group of relatively small (approximately 1 m long) basal coelurosaurs from the Late Jurassic to Early Cretaceous of Europe and Asia. However, Jehol compsognathids such as *Huaxiagnathus* and *Sinocalliopteryx* attained significantly larger sizes, reaching lengths of up to 2.3 m in the latter [4]. In their original description of *Sinocalliopteryx gigas*, Ji et al. [4] commented on the partial leg of an unidentified dromaeosaurid in the posterior region of the abdominal cavity, which they cite as evidence of a highly predaceous lifestyle in *Sinocalliopteryx*. The purpose of this paper is to describe and reassess the abdominal contents of *S. gigas* based on the holotype and a second specimen that indicates wider dietary preferences with implications for feeding strategies of Compsognathidae.

## Materials

**Figure 1:**
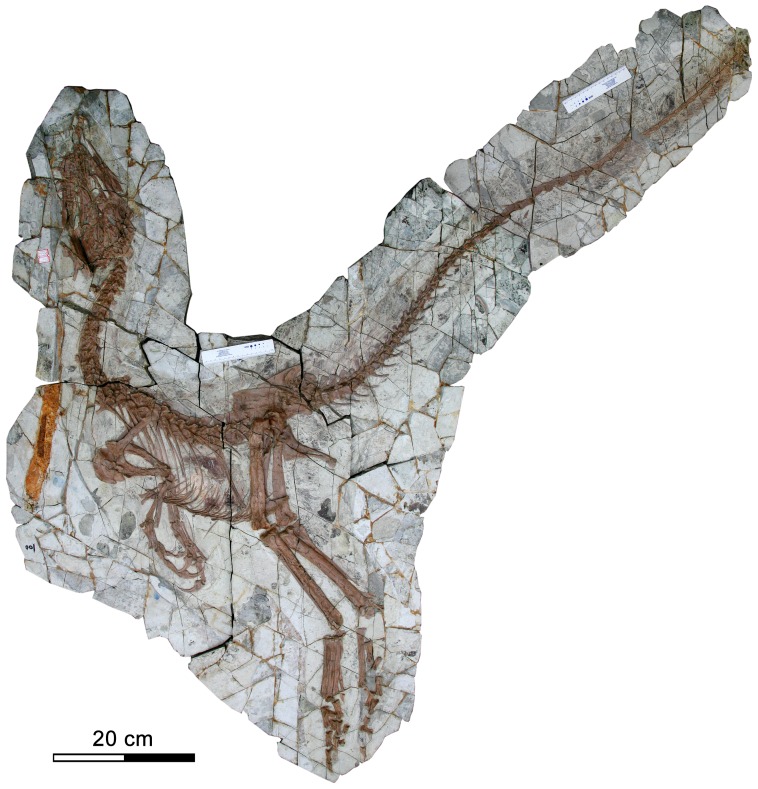
Holotype of *Sinocalliopteryx gigas* (JMP-V-05-8-01).

The holotype of *Sinocalliopteryx gigas* (JMP-V-05-8-01) is a complete, articulated, and exceptionally well-preserved skull and skeleton with long filamentous integument (Figure 1; [4]). A new specimen of *Sinocalliopteryx* sp. (CAGS-IG-T1) is a partially articulated but incomplete skeleton lacking the cervical vertebrae, parts of the dorsal and caudal series, both pectoral and pelvic girdles and the proximal parts of both fore- and hindlimbs. Both specimens are from the Jianshangou Beds of the Yixian Formation (Neocomian; [5], [6]), Beipiao, western Liaoning Province, northeastern China.

### Institutional Abbreviations

**CAGS-IG**, Institute of Geology, Chinese Academy of Geological Sciences, Beijing; China; **JMP**, Jinzhou Museum of Paleontology, Jinzhou, Liaoning Province, China; **NIGP**, Nanjing Institute of Geology and Paleontology, Nanjing, China; **GMV**, China National Geological Museum, Beijing, China.

## Results

### *Sinocalliopteryx* (CAGS-IG-T1) Description and Comparison

**Figure 2:**
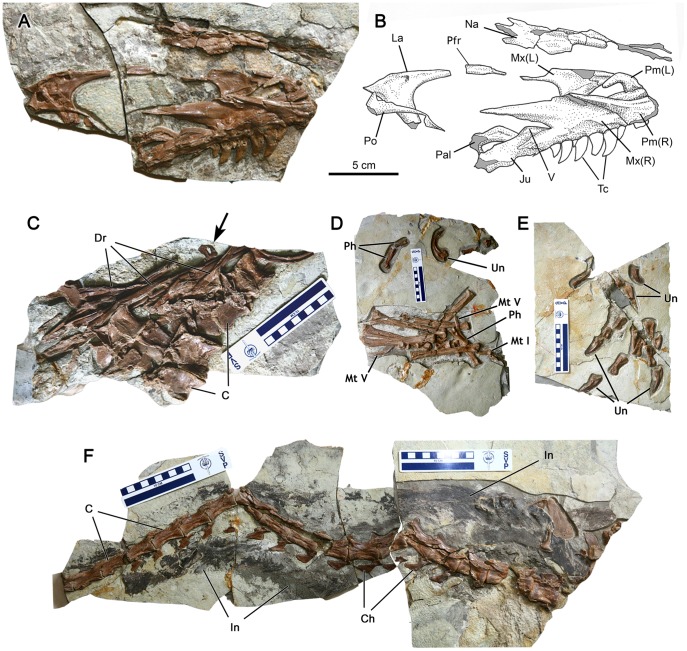
*Sinocalliopteryx gigas* (CAGS-IG-T1), partial skeleton. A, B; skull; C, dorsal vertebrae and ribs. Arrow points to partly covered *Confuciusornis* humerus; D, associated feet; E, associated pedal phalanges and unguals; F, articulated tail with filamentous integument. Abbreviations: C, centrum; Ch, chevron; Dr, dorsal rib; In, integument; Ju; jugal; La, lacrimal; Mt, metatarsal; Mx, maxilla; Na, nasal; Pal, palatine; Pfr, prefrontal; Ph, phalanx; Pm, premaxilla; Po, postorbital; Tc, tooth crown; Un, ungual; V, vomer. Scale bars in C–F equal 10 cm in 1 cm increments.

The skull of CAGS-IG-T1 includes both maxillae, right nasal, right lacrimal, right prefrontal, right jugal, left palatine and vomer, and fragmentary right dentary (Figure 2A, B). The left maxilla, shown in medial view, has at least ten alveoli, six of which hold teeth. Given that the anterior ramus is incomplete, the maxillary tooth count probably exceeded ten by one or two. The most posterior maxillary alveolus is ventral to or slightly anterior to the anterior end of the maxillary-lacrimal contact. The anterior ramus of the maxilla is demarcated by the transition to the posterodorsally-oriented ascending ramus. The posterodorsal process of the maxilla is dorsoventrally deeper than the horizontal ramus. Near the posterior end, the posterodorsal process splits into the larger and longer lateral prong and the smaller and shorter medial prong, between which the lacrimal was clasped. The medial surface of the maxilla above the palatal shelf is smooth and not excavated into the conspicuous maxillary antrum and promaxillary recess as in other theropods [7]. The antorbital fossa has a distinct margin. The maxillary fenestra is absent.

A lacrimal duct is present dorsal to the anterior margin of the preorbital bar. The dorsal edge of the lacrimal is inflated into a longitudinal, dorsally low cornual process. The prefrontal is as long as the anterior ramus of the lacrimal. The postorbital process of the right jugal was anteriorly displaced and now sits on the right and left maxillae. The process retains a groove along its anterior margin that would have received the postorbital. The vomer is dorsoventrally deeper than the horizontal ramus of the maxilla and has a dorsally convex margin. The palatine contacts the maxilla posterior to the maxillary tooth row and posterior to the anterior margin of the antorbital fenestra. The dentary is preserved for the anteroposterior length of three tooth positions.

**Figure 3:**
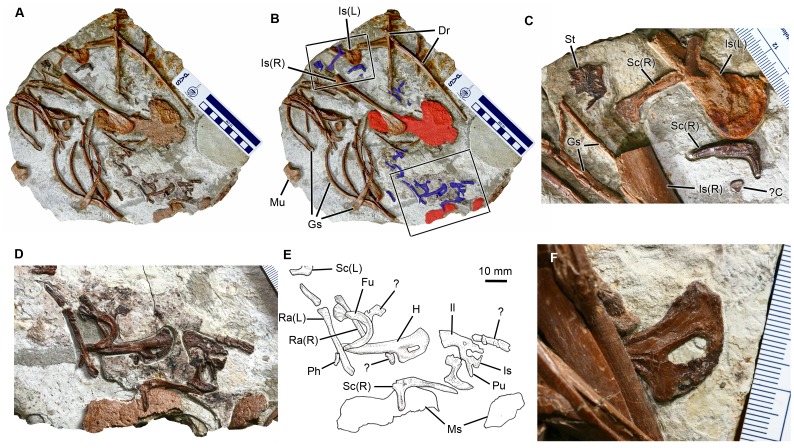
Abdominal contents of *Sinocalliopteryx gigas* (CAGS-IG-T1). A, B; block containing *Confuciusornis* (blue) and unidentified ornithischian (red) remains. C, Close up of *Confuciusornis* sternal and pectoral elements (small box in B); D, E; associated skeleton of *Confuciusornis* (large box in B); F, proximal *Confuciusornis* humerus (arrow in Figure 2). Abbreviations: C, carpal; Dr, dorsal rib; Fu, furcula; Gs, gastralia; H, humerus; Il, ilium; Is, ischium; Mu, manual ungula; Ms, miscellaneous ornithischian bone; Ph, phalanx; Pu, pubis; Ra, radius; Sc, scapulocoracoid; St, sternum. Scale bars in A, B equal 10 cm in 1 cm increments; C, F in 1 mm increments.

Six dorsal vertebrae are preserved in one of the slabs (Figure 2C). The neurocentral sutures are visible in all of these vertebrae, but the sutures are not completely open because the neural arches and the centra are tightly knit. All of the dorsal vertebrae lack pleurocoels as in other compsognathids [8]–[14]. Six left dorsal ribs are preserved with the vertebral series. In a separate slab, two dorsal ribs, nine medial gastralia, and at least ten lateral gastralia surround the abdominal contents of this specimen. The abdominal contents are between the dorsal ribs and the gastralia and partially overlapped by these elements (Figure 3A–F). The right and left ischia as well as the abdomen of *Sinocalliopteryx* CAGS-IG-T1 (along with the gastralia and the abdominal contents) have all shifted posteriorly relative to their position in life. Two elements of the abdominal contents (scapulocoracoid and sternum) lie on a horizontal plane between the left and right ischia (Figure 3C). Two slabs contain caudal vertebrae (Figure 2F). One of the two slabs contains the 11^th^ to 15^th^ caudal vertebrae with L-shaped haemal arches. The other slab contains an articulated series of 13 mid- to distal-caudal vertebrae, of which 11 are entirely preserved. In that slab, only the first two vertebrae have dorsoventrally low neural spines. In comparison with the holotype of *Sinocalliopteryx* [4], the most anterior vertebra in the series represents the 16th caudal vertebra. All but the last two of the vertebrae are associated with L-shaped haemal arches. In the same slab, filamentous integument is preserved along both the dorsal and ventral margins of the tail (Figure 2F). The qualities of preservation and preparation on the specimen do not permit microscopic comparison of the integument. The neurocentral sutures are closed in all mid- to distal-caudal vertebrae.

The forelimb elements are scattered across two slabs. The main forelimb slab has the partially articulated right forearm and hand. The radius, metacarpal II, metacarpal III, and manual phalanx I-1 are complete, whereas other manual elements are overlain on one another such that identification is difficult. Metacarpal III is less than half as wide transversely as metacarpal II. This is the case in *Compsognathus* and *Sinocalliopteryx* [4], [14], [16], but differs from *Huaxiagnathus*, *Nqwebasaurus*, and *Sinosauropteryx*, in which metacarpal II is half as wide transversely as metacarpal III [10], [11], [13]. Although the full length of metacarpal II cannot be measured, it is as long as or slightly longer than manual phalanx I-1, as in *Compsognathus, Huaxiagnathus*, *Juravenator*, *Scipionyx*, and the holotype of *Sinocalliopteryx* [4], [9], [13], [14], [16], [17] but not as in *Sinosauropteryx* in which manual phalanx I-1 is substantially longer [10]. Manual phalanges II-1 and II-2 and the ungual for the digit are preserved near the metatarsals in a separate slab.

Both right and left metatarsals are preserved in a single slab. All of the metatarsals are present for the left foot, whereas the right foot is represented by only metatarsals II–IV (Figure 2D). In the left foot, metatarsal I is 24% of the length of metatarsal III. Metatarsal V is reduced to a curved splint less than half the length of metatarsal IV. Metatarsals II, III, and IV are cylindrical and straight. Distal to the metatarsals is a complete digit III, and two phalanges of digit I. Additional pedal phalanges are in the distal foot slab. The right pedal phalanges II-1, II-2, III-1, III-2, III-3, IV-3, IV-4, and pedal unguals II–IV are present (Figure 2E).

CAGS-IG-T1 clearly represents a compsognathid, distinguished by the nasal excluded from the antorbital fenestra by the maxilla and lacrimal, the absence of pleurocoelus in the dorsal vertebrae, and the manual phalanx I-1 nearly as long as metacarpal II [7], [10], [12], [14], [18]. CAGS-IG-T1 is anatomically almost identical to JMP-V-05-8-01 (the holotype of *Sinocalliopteryx gigas*; [4]) and therefore referable to *Sinocalliopteryx gigas*. CAGS-IG-T1 is larger based on the postcranial measurements (Table 2). The size difference between the two specimens is relatively greater in the length of the metatarsals than in the radius or height of the maxilla, presumably due to allometric growth. Although the original diagnosis of *Sinocallipteryx* does not include any characters preserved in CAGS-IG-T1, this specimen and the holotype of *Sinocalliopteryx gigas* can be distinguished from the similarly-sized, contemporaneous compsognathid *Huaxiagnathus* [13] based on several features of the maxilla: 1) The maxilla not as tall dorsoventrally in both specimens of *Sinocalliopteryx* as it is in *Huaxiagnathus*, in which the maxilla is two thirds taller at maximum than the anterior ramus; 2) The dorsal margin of the posterodorsal process of the maxilla forms an acute angle with the alveolar margin in *Sinocalliopteryx* whereas in *Huaxiagnathus*, the dorsal margin of the process is subparallel to the alveolar margin; 3) The maxillary fenestra is absent in *Sinocalliopteryx*, whereas the fenestra appears to be present in *Huaxiagnathus* [13]; 4) The anterior margin of the antorbital fenestra is dorsal to the seventh or eighth maxillary tooth position in *Huaxiagnathus* [13], whereas the anterior margin of the fenestra is dorsal to at least the ninth or possibly the tenth tooth position in *Sinocalliopteryx*.

**Table 2:** Select measurements (mm) for *Sinocalliopteryx gigas*.

Element	JMP-V-05-8-01	CAGS-IG-T1
Maxillary height (max)	40.8	44.5
Metatarsal III length	147.3	206.3
Radius length	100.7	118.64

### Abdominal Contents of CAGS-IG-T1

A disarticulated but associated skeleton of a confuciusornithine bird is preserved within the posterior part of the *Sinocalliopteryx* abdominal cavityin the vicinity of the distal ends of the ischia and dorsal to the gastral basket (Figure 3A, B). Other remains are scattered throughout the rest of the block. The associated elements include the furcula, left and right scapulocoracoids, right humerus, both radii, a metacarpal II, several manual phalanges, pelvis, a pedal phalanx, possibly part of the femoral shaft, and several unidentifiable bone fragments (Figure 3D, E). The proximal end of a second humerus is preserved in the block containing the dorsal vertebrae, whereas the humerus is partly covered by the dorsal ribs (Figures 2C, 3F). Several additional elements reside in the region between the ischia of *Sinocalliopteryx*, including a dorsal vertebra, at least one phalanx, and part of the shaft of the ?tibiotarsus. The sternum, a ?carpal, and a second right scapulocoracoid are preserved close to the left ischium (Figure 3C). A fourth scapulocoracoid overlies the left ischium.

The furcula, visible in posterior view, is robust and U-shaped. A groove on this posterior surface is typical of *C. santus* [18]. Four scapulocoracoids indicate the presence of at least two individuals. The scapula and coracoid are fused, a condition restricted among Mesozoic Aves to *Confuciusornis sanctus* and *Archaeopteryx lithographica* [18], [19] but also present in some nonavian theropods such as *Velociraptor* [20]. The sternum is damaged, presumably as a result of digestive processes, but retains a median carina as in *C. sanctus*, whereas a carina is absent in *Changchengornis* [19]. The sternum of *Eoconfuciusornis* apparently did not ossify [21].

The humerus is characteristically confuciusornithine, having an expanded proximal end and a triangular deltopectoral crest that constitutes more than one-third of the length of the humerus [19]. An oval foramen pierces the deltopectoral crest, which is an autapomorphic feature of *Confuciusornis* [19], [22]. A deltopectoral foramen is absent in all other confuciusornithids including *Eoconfuciusornis* [21] and *Changchengornis* [23].

The postacetabular process of the ilium is shorter than the preacetabular process and tapers distally. There is no evidence of a brevis fossa, which is present in maniraptoran theropods [19]. The distal end of the ischium is missing, but the proximal portion retains a dorsal process that extends towards but does not contact the postacetabular blade (Figure 3D, E). This feature is present in *C. sanctus* and some enantiornithine birds, but is less developed in *Archaeopteryx* [18], [19].

Based on the aforementioned shared features, the avian remains in CAGS-IG-T1 are unequivocally assignable to *Confuciusornis. Confuciusornis* has had a troubled taxonomic history because the description of the type species was inadequate, and as many as five species have been assigned to that genus. Recent studies, however, have demonstrated that all of these specimens fall within the range of variation for the type species and are therefore synonymous with *C. sanctus* [19], [24]. Moreover, a second genus of confuciusornithid, *Jinzhouornis*, and its two constituent species, has also been shown to be qualitatively and quantitatively indistinguishable from *C. sanctus* [25]. In light of this and the morphological considerations already discussed, the associated confuciusornithid remains in CAGS-IG-T1 are assigned to *C. sanctus.*

**Figure 4:**
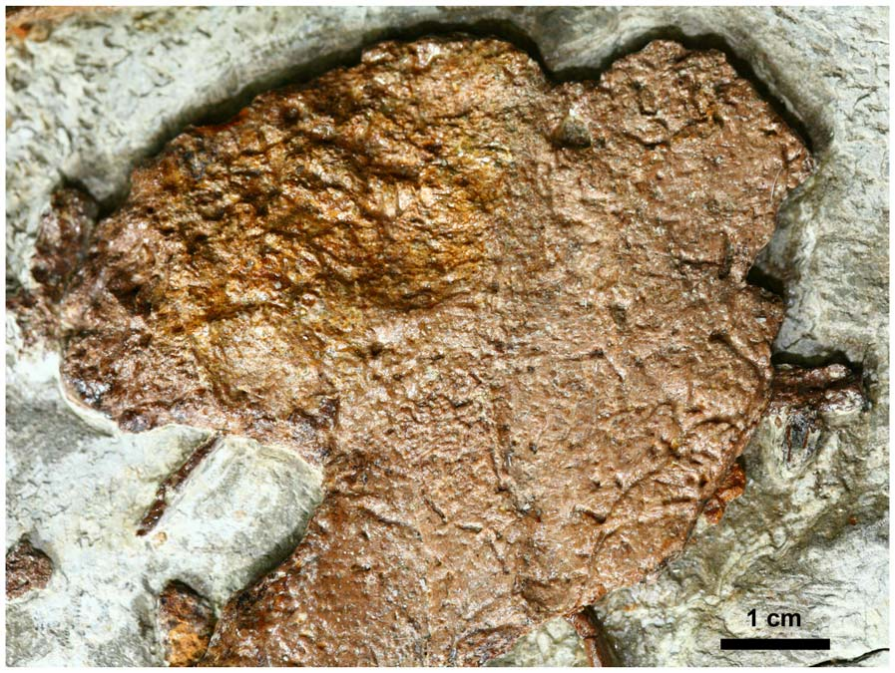
Possible ornithischian scapula (central red element in **Figure 3B**) within the abdomen of *Sinocalliopteryx gigas* (CAGS-IG-T1). Note disorganized bone texture as a result of corrosion by gastric juices.

In addition to confuciusornithid remains, parts of two large bones are also present within the *Sinocalliopteryx* abdominal cavity (Figure 3A, B). Both are platy and incomplete with significant surficial modification; the entire surfaces of both elements are deeply pockmarked, resulting in the almost total loss of the original external bone surfaces (Figure 4). The largest bone, tentatively identified as a scapula, measures 13.5 cm in maximum length and is 6.5 cm wide. The proximal end is expanded dorsoventrally, but the acromion process is incomplete. The anteroventral expansion is larger than the acromion and retroverted such that the posterior margin of the expansion forms an acute angle with the scapula blade. In its short, robust morphology, the scapula resembles the scapulae of *Psittacosaurus* [26] and the basal ornithopod *Yueosaurus* [27]; however, the element is so heavily modified that assignment to any one taxon is contentious.

### Abdominal Contents of JMP-V-05-8-01

**Figure 5:**
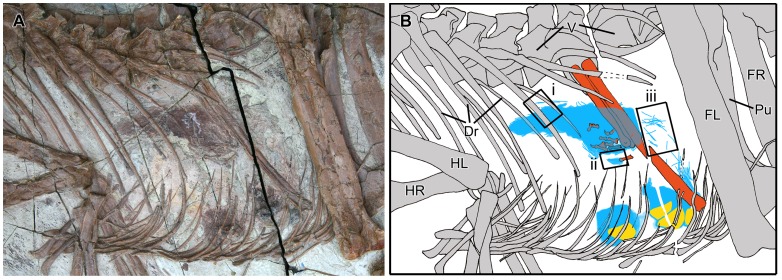
Abdominal contents of *Sinocalliopteryx gigas* (JMP-V-05-8-01). Blue, undigested feather-like structures; Red, dromaeosaurid hindlimb; Yellow, gastroliths. Greek numerals (i–iii) denote enlargements in Figure 5. Abbreviations: Dr, dorsal rib; F, femur; H, humerus; Pu, pubis.

**Figure 6:**
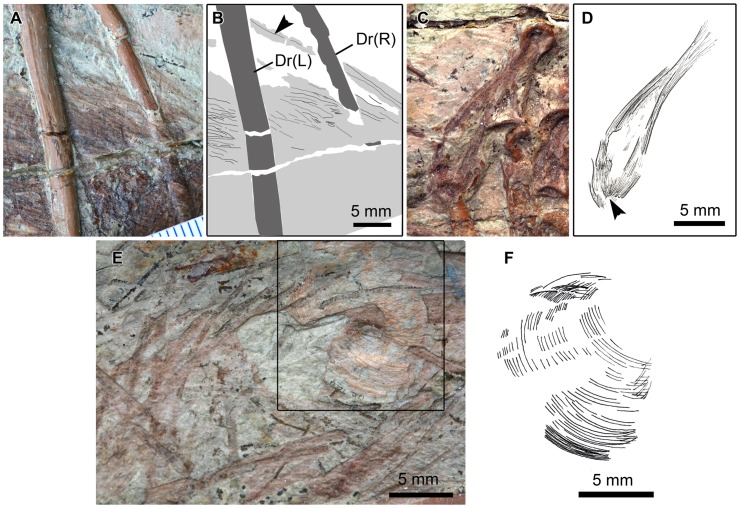
Undigested filamentous integument within the abdominal cavity of *Sinocalliopteryx gigas* (JMP-V-05-8-01). A, B; Enlargement of *i* in Figure 4. Filamentous integument showing central vein (arrow in A; grey lines in B). Black lines are margins between adjacent filaments. Note the stomach contents overlie the right dorsal rib (DrR) but are overlain by the left dorsal rib (DrL). C, D; Enlargement of *ii* in Figure 4. Tuft of filaments showing single point of origin (arrow). E; Enlargement of *iii* in Figure 4 showing scattered filamentous structures. Boxed area and interpretive illustration (F) shows a discrete association of parallel filaments similar to the barbs of an avian feather. Scale  = 5 mm.

The abdominal contents of JMP-V-05-8-01 resembles an inverted C-shape. Forming the upper part of the ‘C’ is a large oval mass (approx. 10 cm long), centrally and dorsally positioned within the abdominal cavity (Figure 5). This mass is composed of a dense accumulation of filamentous feather-like structures up to (and possibly exceeding) 22 mm in length. Where they are less densely gathered, the feather-like structures show fibers that branch off from a central filament (Figure 6A, B). In one area, a single ‘tuft’ shows individual filaments that converge at their bases (Figure 6C, D) in the same arrangement as the tufted integument described for *Sinornithosaurus* [28]. The dromaeosaurid pes and distal part of the leg transects this mass posteriorly to form the vertical part of the ‘C’. A collection of feather-like structures occurs along the length of, but apparently is not connected to, the dromaeosaurid tibiotarsus. A central filament, or rachis, is visible in each of these structures. A discretely arcing arrangement of filaments has a striking resemblance to asymmetrical avian contour feathers (Figure 5E, F). Ventrally, two small, circular masses (approx. 3 cm in diameter) associated with gastroliths [4] are present anterior to the pubic boot. The proximal end of the dromaeosaurid tibiotarsus coincides with the more posterior of the two smaller masses (Figure 5). The two circular masses within the gastral basket are made up of fine, indeterminate matter, with no indication of the filamentous structures seen elsewhere in the gut.

**Figure 7:**
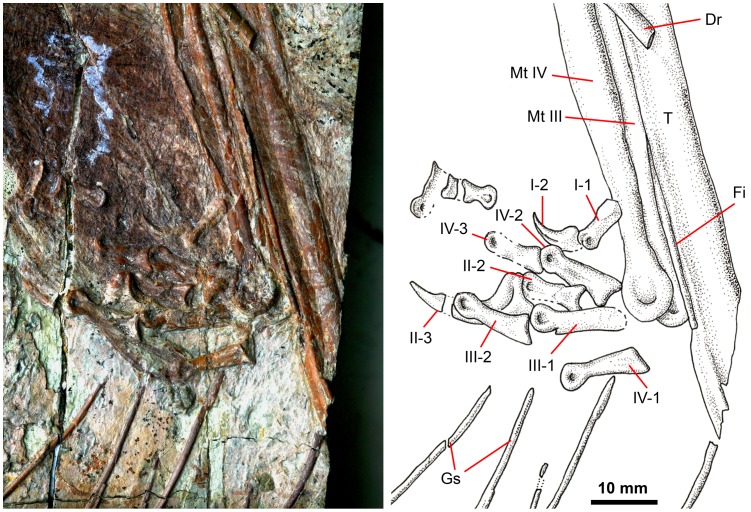
Close up of *Sinornithosaurus* right hindlimb within the abdominal cavity of *Sinocalliopteryx* (JMP-V-05-8-01). Photograph and interpretive illustration. Gastralia (Gs) and dorsal rib (Dr) belong to *Sinocalliopteryx.* Note the similar lengths of metatarsals III and IV. Abbreviations: Fi, fibula; Mt, metatarsal; T, tibia.

The dromaeosaurid hindlimb is from the right side of the body and is preserved with its right lateral side exposed (Figure 5). It is overlain by the left gastralia and the left dorsal ribs and overlays a number of right gastralia and one of the dorsal vertebrae. Therefore, it can be conclusively identified as positioned within the abdominal cavity. The visible limb elements include the tibia, fibula, metatarsals III and IV, and numerous phalanges. Metatarsals I and II are also likely present, but their positions are obscured by matrix and the other limb bones. Some of the phalangeal elements remain articulated, and nearly all the pedal elements are near their articulated positions (Figure 7). The metatarsals lie parallel to the tibia and fibula, with theirproximal ends adjacent to the distal end of the tibia. The phalanges are positioned in a clinched arrangement (Figure 7).

Phalanx II-3 is hypertrophied, which is diagnostic of Dromaeosauridae (Figure 7). As is common among dromaeosaurids from the Jehol Group (including *Graciliraptor, Microraptor, Sinornithosaurus*, and *Tianyuraptor*), the metatarsals are greatly elongate relative to the length of the tibia and fibula [29]–[31], and the shaft of phalanx II-2 is not strongly constricted between the articular facets [29]. Metatarsal IV displays a prominent ventral flange. The metatarsals are semi-arctometarsalian to a greater extent than in *Tianyuraptor* [31]. Unfortunately, the metatarsals are crushed and obscure one another, making other potentially diagnostic characters difficult or impossible to observe. Phalanx III-1 is not exceptionally elongate or slender as it is in *Graciliraptor* [32]. The limb is distinguishable from *Microraptor* based on its overall larger size (tibial length 15.5 cm) and its lower stratigraphic position; however, other, more diagnostic characters of the femur and pedal unguals are missing in JMP-V-05-8-01. The preserved elements are similar to those of *Sinornithosaurus* [29], and it is to this genus that the limb is tentatively referred.

## Discussion

A wide variety of prey items have been identified within the abdominal cavities of compsognathids. Fish and lepidosaurian reptiles were identified within the exceptionally well-preserved digestive tract of *Scipionyx samniticus* [33], the remains of a lepidosaur (*Bavarisaurus* cf. *macrodactylus*) were found within the holotype of *Compsognathus longipes* [8], and bones of an unidentified small mammal within the holotype of *Sinosauropteryx* [10], [15]. A second *Sinosauropteryx* specimen (GMV 2124) preserves the jaws of triconodont (*Sinobaatar*) and symmetrodont (*Zhangheotherium*) mammals [34]. Miscellaneous, partially-digested bones were also observed within the holotype specimen of *Huaxiagnathus* [13]. Based on our identification, the *Sinornithosaurus* limb in *Sinocalliopteryx* (CAGS-IG-T1) corresponds to an individual that can be estimated at roughly one meter in total length [29]. If the *Sinornithosaurus* was predated upon (rather than scavenged), this would imply *Sinocalliopteryx* was capable of tackling carnivorous prey more than a third its own size. The addition of at least two confuciusornithines and an unidentified ornithischian within the abdominal cavity of CAGS-IG-T1 demonstrates a diverse diet in *Sinocalliopteryx.*

**Figure 8:**
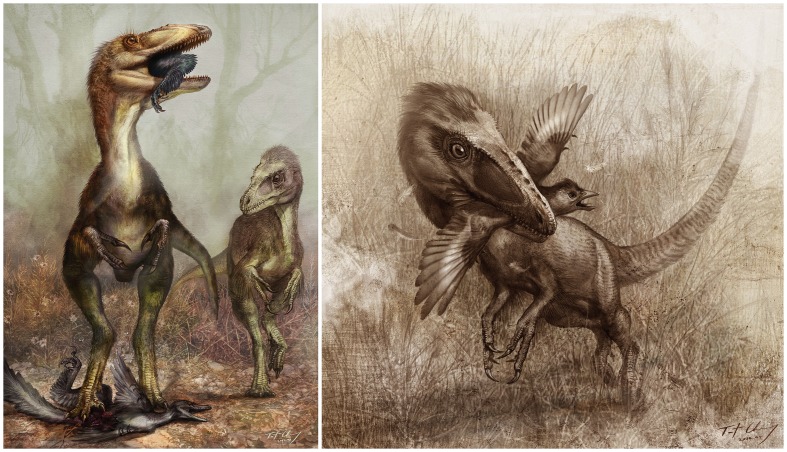
*Sinocalliopteryx* as a stealth hunter feeding on the dromaeosaur *Sinornithosaurus* (left) and the primitive bird *Confuciusornis* (right). Illustration by Cheung Chungtat.

CAGS-IG-T1 possesses abdominal contents in different stages of digestion. The remains of the confuciusornithines, although disarticulated and often broken, still retain relatively smooth (uncorroded) bone surfaces, indicating minimal impact from gastric acids. In contrast, the larger ornithischian bones show considerable corrosive effects and the near total loss of smooth periosteal bone. The marked disparity in digestion (corrosion) between remains indicates a hiatus between the consumption of the ornithischian and subsequent feeding on confuciusornithines.

Among the abdominal contents, several confuciusornithine skeletal elements are notably absent (e.g. skull, ribs, vertebrae, synsacrum, tarsometatarsus). It is unclear if these missing body parts were: 1) never consumed by the *Sinocalliopteryx*; 2) were consumed but were then preferentially dissolved/digested/egested; 3) consumed and preserved but are obscured by matrix and other elements; or 4) are preserved in another block that was not recovered. Barring further preparation and the successful recovery of additional components of the specimen, these competing explanations remain untestable.

### Inferences about the Digestive System of *Sinocalliopteryx*

Information regarding the organs and internal anatomy of dinosaurs is exceptionally rare. Undoubtedly the best example of preserved internal anatomy is that of the juvenile compsognathid, *Scipionyx samniticus* (SBA-SA 163760), which preserves vestiges of many of the major organs in exquisite detail [33], [35]. In addition, remnants of the articular cartilages, ligaments, and muscle tissues are also preserved providing unsurpassed insight into the soft tissue anatomy of a theropod [33]. Moreover, as a compsognathid, *Scipionyx* serves as a useful model for interpreting the abdominal contents and the presumed digestive tract of *Sinocalliopteryx.*

The C-shaped abdominal contents in JMP-V-05-8-01 appear reflective of the original contour of the digestive tract [33], [35]. Furthermore, the contents become smaller and less identifiable along the length of the inferred gut, presumably as a result of more advanced digestion. The largest mass within the abdomen of JMP-V-05-8-01 contains discernible feather-like structures and the partial leg of an ingested dromaeosaurid. These remains most likely represent a cololite that delimits the stomach. Further along the length of the C-shaped digestive tract, the two smaller food masses are composed of amorphous material suggestive of longer residence times within the digestive tract. Their proximity to the stomach suggests they may have been contained within the duodenal loop, which is distinct in *Scipionyx* [33], and modern birds [36]. In *Scipionyx*, the anterior part of the descending loop of the duodenum (i.e. behind the pyloric sphincter) is dorsoventrally oriented. Further along its length, the duodenum turns posteriorly, becoming parallel with the gastral basket in precisely the same way as the abdominal contents of JMP-V-05-8-01. The duodenum of *Scipionyx* also contains incompletely digested elements (lizard-like squamae and a possible fish vertebra [33]), which is consistent with the progression of ingested remains in *Sinocalliopteryx.*

There is evidence that crocodilians can increase secretion of stomach acids by shunting deoxygenated blood to the stomach (by increasing levels of PCO_2_; [37]), giving them the most acidic foregut yet measured in any animal. Gastric pH may drop as low as 1.2 in crocodilians [38], whereas it is generally always above 2.6 in birds [39]. The increase in acidity in crocodilians may also be an adaptation to deal with large meals (*A. mississippiensis* will voluntarily consume 23% of its body mass at one time; [40]). Because of low acidity, most modern birds are unable to digest bone and instead will compact and orally egest this indigestible material [39]. Given the presence of acid-etched bones in the gut of *Sinocalliopteryx* and *Scipionyx*, as well as the presence of undigested bone and muscle fibers in theropod coprolites [41], [42], it is known that at least some carnivorous dinosaurs possessed highly acidic foreguts conducive to digestive processing of bone [35]. Preserved theropod feces from Late Cretaceous tyrannosaurids retain modified bone fragments [41], [42], implying that some ingested bone was not regurgitated in at least some non-avian theropods. However, undigested muscle tissue from a tyrannosaurid coprolite [42] suggests that some non-avian theropod digestive tracts were not as destructive as those of extant crocodilians. Therefore, modern crocodilians do not necessarily provide ideal analogues for *Sinocalliopteryx* digestion. Based on the digestive efficacy of *Alligator mississippiensis* [37], a predicted minimum gastric residence time of 13 days would be required to reach the level of corrosion observed in the presumed ornithischian bones. By comparison, the gastric residence time for birds is generally less than 12 hours [36].

Geo-gastroliths are swallowed sediment particles such as pebbles and grit irrespective of function or deliberate/accidental origin [43]. Such stones are known from a wide variety of theropods including *Allosaurus* [44], *Baryonyx* [45], *Caudipteryx* [46], *Lourinhanosaurus* [47], *Nqwebasaurus* [48], *Sinornithomimus* [49], *Sinosauropteryx* [50], *Syntarsus* [51], and possibly *Tarbosaurus* [52]. In a recent review of geo-gastrolith function, Wings [43] found aid in digestion (trituration, food mixing, stomach cleaning, and mineral supplement) as the most plausible reason for the deliberate ingestion of stomach stones. However, accidental ingestion (e.g. consumption of gastrolith-containing prey) was found to be a major factor in extant carnivores, including crocodilians. The apparent absence of geo-gastroliths in CAGS-IG-T1 suggests such stones were not a critical part of *Sinocalliopteryx* digestion. In fact, Wings [43] argued that low numbers of stomach stones, such as those found in *Allosaurus* [43] and *Baryonyx* [45] are likely the result of accidental ingestion. Discrete accumulations of dozens or hundreds of stones in many individuals, such as those in the ornithomimid *Sinornithomimus* [49], are almost certainly digestion aids. It is therefore likely that the few stones found in the holotype of *Sinocalliopteryx* were a result of accidental ingestion. It is notable that geo-gastroliths in *Sinocalliopteryx* occur in the posterior abdomen rather than the stomach where they occur in extant crocodilians [38]. In crocodilians, a particularly strong pyloric sphincter prevents the passage of geo-gastroliths into the midgut [38], [53]. In birds, geo-gastroliths are held within the muscular ventriculus, or gizzard, which functions as the primary trituration site [53]. In *Sinocalliopteryx*, the association between geo-gastroliths and the highly processed food masses in the midgut region negate the possibility of a gizzard. Had the animal lived, it is likely that these stones would have been passed in the faeces.

### Predation on Flying Prey and Ecological Implications

Remains as delicate as small bird bones have presumably short digestion periods, and the multiple *Confuciusornis* within the abdominal cavity of CAGS-IG-T1 must have been consumed in fairly rapid succession, in order for the first individual not to have had time to be digested noticeably beyond that of the second. Moreover, levels of corrosion on all the confuciusornithine elements are similar on a macroscopic level, again suggesting that the birds were consumed in rapid succession. Such short durations between meals provides anecdotal evidence for high metabolic rate in *Sinocalliopteryx*.

In both CAGS-IG-T1 and JMP-V-05-8-01, scavenging cannot be definitively ruled out as an alternative to predation. However, as argued by O’Connor et al. [54], a high degree of articulation among gut contents shows that, when ingested, the carcasses were at least fresh enough not to have disarticulated. The association of two or more birds is perhaps more easily explained by selective hunting than by the chance discovery of multiple *C. sanctus* carcasses; however, this is speculative. In the case of CAGS-IG-T1, it is improbable that every individual organism represented within the gut contents was consumed exclusively as a result of scavenging, as true obligate tetrapod scavengers are rare [55].

The presence of at least two confuciusornithine birds within the abdominal cavity of *Sinocalliopteryx* (CAGS-IG-T1) argues against circumstantial consumption (i.e. the coincidental scavenging of two or more carcasses of the same species), and suggests a behavioral proficiency for predating on flying prey. It is not known if the dromaeosaurid *Sinornithosaurus* possessed elongate hind and forelimb feathers, as in the closely related *Microraptor*. If it did, the *Sinornithosaurus* remains within JMP-V-05-8-01 may constitute an additional example of a flight-capable maniraptoran eaten by a *Sinocalliopteryx*.

O’Connor et al. [54] reported on a specimen of *Microraptor* with the remains of an enantiornithine bird within its abdominal cavity, and argued that such presumed predation on a bird with clear arboreal perching adaptations was evidence supporting a highly arboreal/aerial lifestyle in *Microraptor*. Based on various other lines of evidence, we agree with this ultimate conclusion; however, that Jehol birds were evidently on the menu of *Sinocalliopteryx* must be regarded as a strong contradiction to the necessity of O’Connor et al’s [54] ecological inference. *Confuciusornis* was not as well adapted to perching as enantiornithine birds, but does nonetheless possess long curved pedal claws and a posteriorly-facing hallux, and was capable of powered flight. While *Sinocalliopteryx* does have proportionately longer arms than most compsognathids and may have been capable of tree climbing, it lacks any definitive arboreal adaptations; at over two meters in length, is best regarded as a predominantly terrestrial animal.

Active hunting of flight-capable prey by a land-bound predator may seem intrinsically implausible, but there are abundant extant examples, wild felids among the most famous. The back-footed cat (*Felis nigripes*) of southern Africa routinely ambushes and chases down cursorial birds before they are able to become airborne [56]. Servals (*Leptailurus serval*) are long-legged and adept at pouncing on alighted birds, and at snagging fleeing birds midair [57]–[59]. Avian prey is known to constitute nearly half the diet of some leopard cats (*Prionailurus bengalensis*) [60], which both climb trees to prey on roosting birds and ambush foraging birds on the ground. Among canids, foxes are expert bird hunters, commonly taking anseriforme, galliforme, and passeriforme game [61], [62]. Among extant reptiles, monitor lizards and various snakes consume birds in both arboreal and terrestrial contexts [63]–[66].

In a majority of these examples, what is required to successfully apprehend avian prey is not climbing prowess, but stealth, such that the predator can reach its striking distance before the prey takes flight. It should be remembered that *Confuciusornis* and other Jehol birds were not as well adapted for flight as most modern aves, and, therefore, likely required greater time to mount an aerial takeoff and escape. Nevertheless, the evidence of bird predation in *Sinocalliopteryx* suggests that it was a highly capable stealth hunter (Figure 8).
